# *Staphylococcus carnosus*: from starter culture to protein engineering platform

**DOI:** 10.1007/s00253-017-8528-6

**Published:** 2017-10-02

**Authors:** John Löfblom, Ralf Rosenstein, Minh-Thu Nguyen, Stefan Ståhl, Friedrich Götz

**Affiliations:** 1 Division of Protein Technology, School of Biotechnology, KTH-Royal Institute of Technology, AlbaNova University Center, Roslagstullsbacken 21, 106 91 Stockholm, Sweden; 2 Microbial Genetics, Interfaculty Institute of Microbiology and Infection Medicine and Infection Medicine (IMIT), University of Tübingen, Auf der Morgenstelle 28, 72076 Tübingen, Germany

**Keywords:** Bacterial surface display, Combinatorial protein engineering, Epitope mapping, Food fermentation, Starter culture, Virulence factors

## Abstract

Since the 1950s, *Staphylococcus carnosus* is used as a starter culture for sausage fermentation where it contributes to food safety, flavor, and a controlled fermentation process. The long experience with *S. carnosus* has shown that it is a harmless and “food grade” species. This was confirmed by the genome sequence of *S. carnosus* TM300 that lacks genes involved in pathogenicity. Since the development of a cloning system in TM300, numerous genes have been cloned, expressed, and characterized and in particular, virulence genes that could be functionally validated in this non-pathogenic strain. A secretion system was developed for production and secretion of industrially important proteins and later modified to also enable display of heterologous proteins on the surface. The display system has been employed for various purposes, such as development of live bacterial delivery vehicles as well as microbial biocatalysts or bioadsorbents for potential environmental or biosensor applications. Recently, this surface display system has been utilized for display of peptide and protein libraries for profiling of protease substrates and for generation of various affinity proteins, e.g., Affibody molecules and scFv antibodies. In addition, by display of fragmented antigen-encoding genes, the surface expression system has been successfully used for epitope mapping of antibodies. Reviews on specific applications of *S. carnosus* have been published earlier, but here we provide a more extensive overview, covering a broad range of areas from food fermentation to sophisticated methods for protein-based drug discovery, which are all based on *S. carnosus*.

## Introduction

This review article is unique in its nature in that it describes the use of the food grade Gram-positive bacterium, *Staphylococcus carnosus*, evolving over several decades, from being an important strain in food fermentation (Götz 1990c) to becoming a versatile and powerful microbial tool in modern microbiology and biotechnology. When the genome sequence was deciphered (Rosenstein and Götz 2010; Rosenstein et al. 2009), the different characteristics of *S. carnosus* were better understood, and as will be described, its non-pathogenic nature made it suitable for characterization of virulence factors. The development of a host-vector system for efficient and secreted recombinant production inspired the development of also a surface display system for *S. carnosus*. The use of these systems in a wide variety of application areas will be reviewed.

## *S. carnosus* as a starter culture

**Fig. 1:**
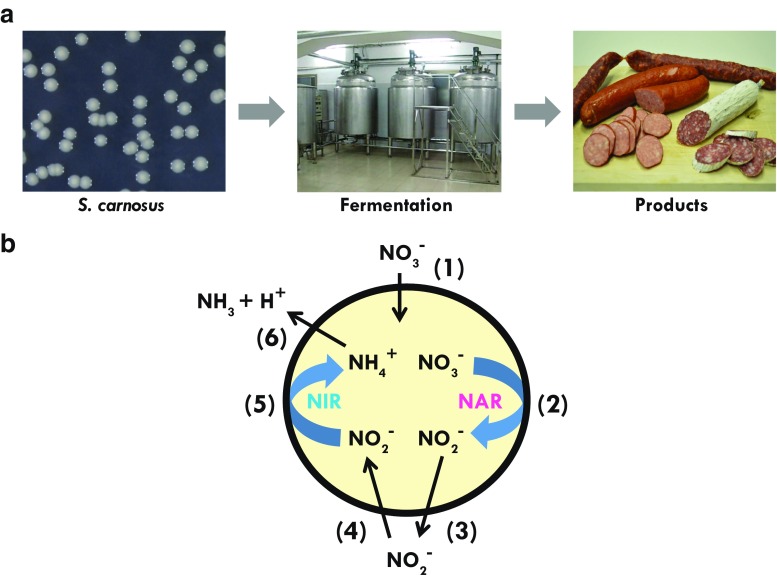
Application of *S. carnosus* in food technology. **a** *S. carnosus* is used as starter culture for sausage fermentation where dissimilatory nitrate/nitrite reduction plays an important role. **b** Steps in dissimilatory nitrate/nitrite reduction in *S. carnosus* under anaerobic conditions. (1) Nitrate is taken up by the nitrate transporter (NarT). (2) It is reduced to nitrite by nitrate reductase. (3) Nitrite is excreted and accumulates in the supernatant until nitrate is almost completely consumed. (4) Nitrite is taken up again and is ((5)) intracellularly reduced to ammonia by the NADH-dependent nitrite reductase. (6) Ammonia is excreted leading to mild alkalization of the environment

Some of the most well-investigated staphylococcal species (e.g., *S. aureus*) are pathogens. However, like many other genera, *Staphylococcus* is composed of many species (> 40) with a vast diversity, of which only few are associated with pathogenicity. The majority has never been associated with infection, and some species are even used as starter cultures in sausage fermentation (Götz et al. 2006). The first reports on using *S. carnosus* in sausage fermentation came in the 1950s (Lerche and Sinell 1955; Niinivaara and Pohja 1956). At that time, they were regarded as micrococci, a group of Gram-positive cocci that are facultative anaerobic and catalase-positive. However, a systematic analysis of the starter cultures in various fermented dry sausages revealed that most of these micrococci were incorrectly classified and are in fact *S. carnosus* (Schleifer and Fischer 1982). *S. carnosus* and *S. xylosus* are the two main staphylococcal species worldwide that are used as starter cultures in food fermentation, either alone or in combination with defined lactobacilli or other microorganisms. Starter cultures protect the food from undesirable bacteria and make the fermentation process more reliable. They also suppress food spoilage and poisoning by unwanted microorganisms and the whole fermentation process can be better controlled. *S. carnosus* has several functions during the ripening process of dry sausage (Barriere and Leroy-Setrin 2001; Corbiere Morot-Bzot et al. 2007; Liepe and Porobic 1983); nitrate is reduced to nitrite which, together with myoglobin, forms the red colored nitrosomyoglobin (Neubauer and Götz 1996; Götz 1990c). Subsequently, nitrite is further reduced to ammonia which leads to regeneration of NAD^+^ that is needed for glycolysis (Neubauer et al. 1999). *S. carnosus* also contributes to flavor and to detoxification of hydrogen peroxide that is produced by lactobacilli (Barriere and Leroy-Setrin 2001). Because of its use as a starter culture since the 1950s, *S. carnosus* is regarded as a “food grade” species (Fig. 1a).

## Dissimilatory nitrate fermentation

Beside flavor, one of the main functions of *S. carnosus* as a starter culture is its ability to reduce nitrate and nitrite. Nitrate and/or nitrite are curing agents that play a decisive role in obtaining the specific sensory properties, stability, and hygienic safety of products such as fermented sausages, ham, and more recently, emulsion type of sausages (Hammes 2012). The intermediary presence of nitrite is important as it prevents the growth of food-spoiling bacteria such as *Clostridium*. On the other hand, at the end of the fermentation process, both nitrate and nitrite should be decreased below a certain threshold level. As many lactobacilli are unable to reduce nitrate, *S. carnosus* has an important function in the process. In *S. carnosus,* the reduction of nitrate to ammonia involves several steps (Fig. 1b) (Neubauer and Götz 1996): (i) nitrate is taken up and reduced to nitrite, and nitrite is subsequently excreted, (ii) after depletion of nitrate, the externally accumulated nitrite is taken up by the cells and reduced to ammonia, which again is excreted into the medium. The nitrate reduction by the nitrate reductase is connected with energy gain and is therefore also referred to as “anaerobic respiration” or “dissimilatory nitrate reduction” (Fast et al. 1996; Fedtke et al. 2002). The nitrate reductase is a membrane-bound enzyme, whereas nitrite reductase is a cytosolic enzyme involved in NADH reoxidation (Neubauer et al. 1999; Pantel et al. 1998). The expression of the corresponding genes is only possible under anaerobic growth conditions and in the presence of nitrate. The mechanism of oxygen repression is based on a three-component system, NreABC (Schlag et al. 2008). NreB is an oxygen-sensing histidine protein kinase with an O-labile iron-sulfur cluster of the FNR type (Kamps et al. 2004; Müllner et al. 2008). NreA functions as a nitrate receptor (Niemann et al. 2014), which together with NreB forms a nitrate-oxygen sensor complex (Nilkens et al. 2014). NreC is phosphorylated by NreB and the phospho-NreC acts as a response regulator that specifically binds to a guanine-cytosine (GC)-rich palindromic sequence to enhance transcription initiation of all operons involved in nitrate/nitrate metabolism (Fedtke et al. 2002).

## Characterization of the genome of *S. carnosus* TM300

**Fig. 2:**
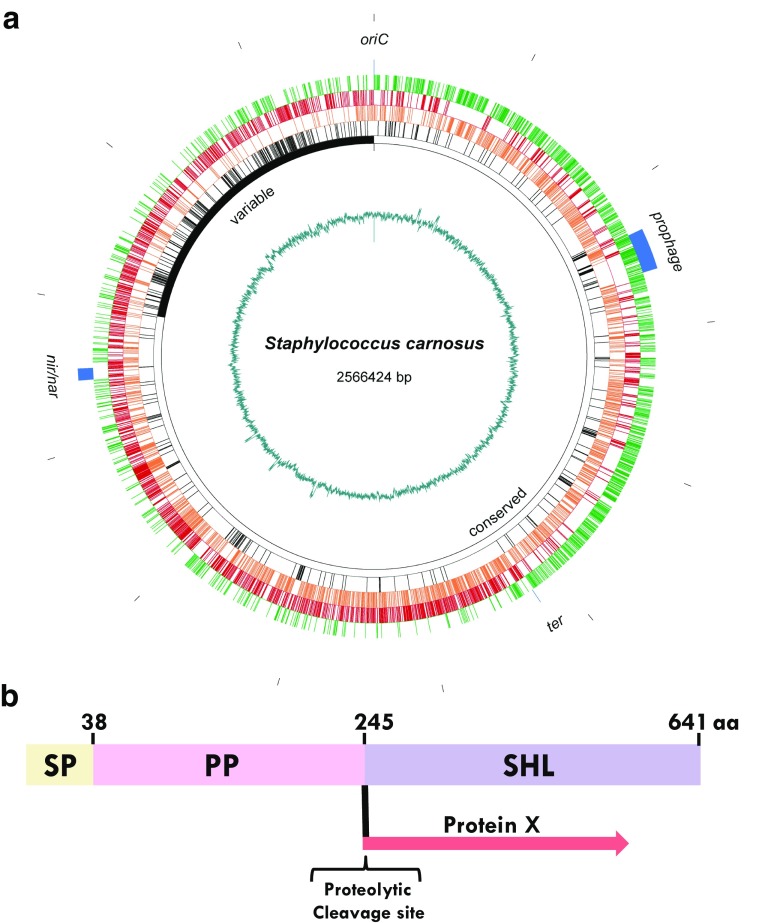
Illustration of *S. carnosus* genomic map and protein construction for secretion. **a** Genomic map of *S. carnosus*. The green circle represents genes located in the upper strand; the red circle indicates genes from the lower strand. Orange dashes show genes that are conserved within the staphylococci, while the black dashes correspond to genes that are specific for *S. carnosus* TM300. The extension of the conserved core region of the genome is shown by the open black circle; the variable region with an accumulation of species-specific genes is located next to the origin of replication (*oriC*) and indicated as filled black segment. Note that the point of termination replication (*ter*) is located asymmetrically with respect to *oriC*. The blue segments show the positions of a prophage and the genes responsible for nitrite and nitrate reduction (*nir*/*nar*), respectively. A GC plot showing local deviations in GC content is presented on the innermost circle. The scale is shown in the outermost circle with the ticks indicating every 0.2 million bases. **b** SHL-based secretion signals for heterologous secretion of proteins (secretion construct). The signal peptide (SP) and the propeptide (PP) of SHL (*Staphylococcus hyicus* lipase) is used to secrete other proteins (protein X) into the supernatant in high amounts. The PP part can be cleaved off by a specific protease that cleaves at the proteolytic cleavage site introduced between PP and protein X. Normally, enzymes are active even in the presence of PP

The 2.56-Mbp genome of *S. carnosus* TM300 is relatively different from other sequenced genomes of this genus. It is small compared with other genomes and it has the highest GC content (34.6%) of all sequenced staphylococcal species (Rosenstein and Götz 2010; Rosenstein et al. 2009). Another peculiarity is that the *ori* and *ter* regions are asymmetrically arranged with the replichores I (1.05 Mbp) and II (1.5 Mbp) (Fig. 2a). Such an asymmetry could have arisen by a large deletion near the oriC. Our experience with gene cloning and expression in *S. carnosus* showed that we normally have no trouble with genetic instability. This positive quality could be due to the absence of mobile elements such as plasmids, IS elements, transposons, or STAR elements. Furthermore, the number of repeat sequences has markedly decreased suggesting a comparatively high stability of the genome. In comparison, *S. aureus* and *S. epidermidis* strains have numerous such elements and repeat sequences.

Genome analysis revealed that the main pathways are present, although some genes are truncated. This is probably due to the nutrient-rich habitat, which makes some biosynthesis functions superfluous. The latter is particularly important for the bacteria to tolerate the high osmolality in sausage meat. The genome also lacks most of the toxins typical of *S. aureus* as well as genes involved in biofilm formation and adherence to host cells and matrix proteins (Rosenstein and Götz 2010; Rosenstein et al. 2009). While pathogenic species such as *S. aureus* are completely resistant to lysozyme, *S. carnosus* and other non-pathogenic species are sensitive to lysozyme, which is produced by mammalians in response to a bacterial infection. The reason for the high lysozyme resistance in *S. aureus* is due to the presence of the peptidoglycan O-acetyltransferase (OatA) that modifies the peptidoglycan in such a way that lysozyme binding is affected (Bera et al. 2005). *S. carnosus* and other non-pathogenic species lack the *oatA* gene (Bera et al. 2006). In conclusion, the lack of toxins, hemolysins, many of the adherence proteins, capsule genes, the presence of an unusual high number of truncated genes, and the lack of the peptidoglycan O-acetyltransferase (OatA) underscore the non-pathogenic status of *S. carnosus*.

The natural habitat of *S. carnosus* is still not known today and it has never been associated with pathogenicity. However, its close phylogenetic relationship with *S. piscifermentans*, which is associated with marine fish, suggests that *S. carnosus* comes from a similar biotope (Probst et al. 1998; Tanasupawat et al. 1992). Although the ecological niche of *S. carnosus* and its related species is unclear, we assume that *S. carnosus* is well equipped to live in a milieu together with Gram-negative bacteria such as *Pseudomonas*. For example, co-cultivation studies of *S. carnosus* with *Pseudomonas aeruginosa*, an opportunistic pathogen, revealed that *P. aeruginosa* was unable to suppress the growth of *S. carnosus*, but it massively suppressed the growth of *S. aureus*. *P. aeruginosa* and related species produce a number of respiratory inhibitors like pyocyanin (Hassan and Fridovich 1980), hydrogen cyanide (Castric 1975), and a mixture of quinoline *N*-oxides (Machan et al. 1992). While *S. aureus*, *S. epidermidis*, or *S. saprophyticus* are sensitive to these respiratory inhibitors, *S. carnosus* and *S. piscifermentans* are resistant (Voggu et al. 2006). The resistance is due to the *cydAB* genes that encode a pyocyanin and cyanide resistant cytochrome *bd* quinol oxidase. In *S. aureus* and other pathogenic species, the cytochrome *bd* quinol oxidase does not cause resistance (Voggu et al. 2006). It has been shown that in *S. aureus*, the subunit B was altered in such a way that it became sensitive. We assume that *S. aureus* and other pathogenic staphylococcal species rarely come in contact with *Pseudomonas* and that they have lost the cyanide resistance function by successive mutations in the *cydB* gene, a process referred to as “micro evolution” (Voggu et al. 2006). The *cydAB* operon is also found in *Escherichia coli* where it is referred to as a cytochrome *d* oxidase complex, which is particularly active under oxygen limited conditions (Cotter et al. 1997). We assume that *S. carnosus* and related species live in an environment that is also occupied by *Pseudomonas* and other Gram-negative bacteria and that they have evolved to resist cyanide and pyocyanin to be able to co-exist with *Pseudomonas*. As for *S. aureus*, there was probably no need to compete with *Pseudomonas*, and the *cydB* gene was degenerated to cyanide-sensitive respiration.

## *S. carnosus* as a valuable tool to analyze virulence functions

The lack of most virulence factors makes *S. carnosus* a suitable model organism to study pathogenicity factors from pathogenic staphylococcal species. Numerous invasion factors and matrix-binding proteins have been expressed in *S. carnosus*, to unravel and to prove their functions. For example, unlike *S. aureus*, *S. carnosus* has no fibronectin-binding proteins and therefore, the function of heterologous-expressed proteins and their role in binding to other matrix proteins or role in host cell invasion has been verified and studied in *S. carnosus* (Agerer et al. 2005; Grundmeier et al. 2004; Kerdudou et al. 2006; Sinha et al. 2000). The proof that the extracellular adherence protein (Eap) from *S. aureus* enhances host cell internalization was carried out in *S. carnosus* (Haggar et al. 2003). The broad-spectrum binding capacity of the *S. aureus* extracellular matrix protein-binding protein (Emp) was verified in *S. carnosus* (Hussain et al. 2001). The proof that the peptidoglycan O-acetyltransferase (OatA) causes lysozyme resistance was made in *S. carnosus* because transformation of the *oatA* gene into *S. carnosus* rendered the clones lysozyme resistant (Bera et al. 2006). The finding that the *S. aureus*-specific *lpl* gene cluster triggers host cell invasion was supported by transforming the *lpl* gene cluster into the non-invasive *S. carnosus* which became invasive after receiving the gene cluster (Nguyen et al. 2015). Also, *S. epidermidis*-derived virulence factors have been studied and verified in *S. carnosus*, such as phenol-soluble modulin peptides (Otto et al. 2004), methicillin resistance gene (Tesch et al. 1988), and biofilm formation of the *S. epidermidis*-derived *ica* genes (Heilmann et al. 1996, 2004).

## Development of a cloning and protein production system in *S. carnosus*

Because of its long use in starter cultures for meat fermentation, *S. carnosus* is classified as a GRAS (generally recognized as safe) organism and a cloning system has therefore been developed for this species. When used as a cloning and production host, it is necessary that it can be transformed with recombinant DNA, that vectors are stably replicated, and that it has low extracellular protease activity to prevent proteolytic degradation of secreted recombinant proteins. Almost 100 *S. carnosus* strains were screened for transformation ability and lack of external proteolytic activity. Among those strains, *S. carnosus* TM300 was superior and therefore selected as a potential cloning and protein production host (Götz 1990c). Indeed, TM300 does not secrete soluble exoproteases, lipases, or hemolysins into the culture medium.

The first efforts focused on developing an efficient plasmid transformation method. Initially, the method of choice was protoplast transformation (Götz et al. 1983a), which was later improved to increase the transformation frequency (Götz and Schumacher 1987). Protoplast transformation is relatively laborious, but the reached efficiency was 10^6^ transformants per μg DNA. With the advent of the less time-consuming electroporation, this method was soon applied successfully to *S. carnosus* (Augustin and Götz 1990) and was later optimized by Löfblom and coworkers (Löfblom et al. 2007a). Plasmids are widely distributed in staphylococci and some classical plasmids such as pT181, pC194, and pSX297 (Götz et al. 1983b; Horinouchi and Weisblum 1982; Novick et al. 1982) served as a basis for vector construction such as pCT20 and pCA43 (Keller et al. 1983; Kreutz and Götz 1984) or the xylose inducible and glucose repressible vectors pTX15 and pCX15 (Peschel et al. 1996; Wieland et al. 1995). In the meantime, optimized derivatives of these vectors were generated. There was also a gene replacement system developed in *S. carnosus* and *S. xylosus* that was based on temperature-sensitive *Escherichia coli-Staphylococcus* shuttle vectors for fragment delivery and erythromycin resistance cassettes to facilitate selection of genomic copies of disrupted genes (Brückner 1997). With the development of these basic tools, a number of genes could be cloned, expressed, and analyzed for function in *S. carnosus* (Brückner and Götz 1995; Götz 1990a, 1990b, 1990c).

## *Staphylococcus hyicus* lipase (SHL)-based construct for secretion of proteins

The lipase gene (*lip*) from *Staphylococcus hyicus* subsp. *hyicus* was one of the first genes that was subcloned in *S. carnosus* (Götz et al. 1985). The *lip*-encoded lipase was named SHL (*S. hyicus* lipase) (Rosenstein and Götz 2000). SHL is the most well-characterized lipase among the staphylococcal lipases. Its activity is Ca^2+^-dependent, and the enzyme should rather be regarded as a phospholipase as its activity with phospholipids was higher than with triglycerides (van Oort et al. 1989). Triglycerides were fully hydrolyzed to free fatty acid and glycerol and the fatty acids of phosphatidylcholines and lysophospholipids were also completely hydrolyzed. Thus, SHL is unique among staphylococcal lipases as it has both lipase and an even higher phospholipase A1 and lysophospholipase activity. Structural analysis of the mature SHL showed that the substrate-binding cavity contains two large hydrophobic acyl chain-binding pockets and a shallow and more polar third pocket that is capable of binding either a short fatty acid or a phospholipid head group, explaining the broad substrate specificity (Tiesinga et al. 2007).

SHL turned out to be a paradigm of staphylococcal lipases as all the lipases studied so far are organized as pre-pro-lipases (Götz and Rosenstein 2001; Rosenstein and Götz 2000). The pre-sequence represents the signal peptide, which is unique as it contains a conserved YSIRK-G/S motif which appears to be involved in enhanced protein translocation or processing (Bae and Schneewind 2003; Rosenstein and Götz 2000). The 207 amino acid long propeptide (PP) is located between the signal peptide (SP) and the mature part of the lipases. Normally, the lipases are secreted in the pro-form, which is subsequently processed by an extracellular protease (Götz et al. 1998; Wenzig et al. 1990). In *S. aureus*, the processing enzyme is the metalloprotease aureolysin (Cadieux et al. 2014). Complete or partial deletion of the PP dramatically impaired signal peptide processing, secretion, and lipase stability, suggesting that the PP acts as an intramolecular chaperone (Demleitner and Götz 1994; Liebl and Götz 1986). The PP also protected the *Escherichia coli* outer membrane protein A (OmpA) from proteolytic degradation by cell-associated protease(s) in *Bacillus subtilis* (Meens et al. 1997).

Both the SHL-specific SP and PP were necessary to secrete heterologous proteins in large amounts (Fig. 2b). For example, the human growth hormone protein (hGH) was efficiently produced by *S. carnosus* when fused with the PP, which can be removed from hGH by introducing an enterokinase cleavage site between PP and hGH (Sturmfels et al. 2001). In a pH-auxostatic fed-batch process, the production of the human calcitonin (hCT) precursor fusion protein reached a concentration of 2000 mg/L within 14 h, and after cleavage of the PP, still 420 mg/L of the recombinant hCT precursor was obtained (Dilsen et al. 2000, 2001). SHL production could be increased up to 230 mg/mL by specific fermentation techniques (Lechner et al. 1988; Märkl et al. 1990). The SHL-specific SP and PP were also successfully used to secrete large amounts of the *Escherichia coli*-specific alkaline phosphatase (phoA) in *Bacillus subtilis*; the PP protected the target protein from proteolytic degradation in the *B. subtilis* supernatant (Kouwen et al. 2010). Thus, the SHL-PP not only contributes to folding and secretion but also protects the fusion partner from proteolytic degradation. This system was also used for immobilization of enzymatically active enzymes on the cell surface of *S. carnosus* (Strauss and Götz 1996). These and many other examples show that the SHL secretion signals comprise a very valuable biotechnological tool for protein production/secretion in *S. carnosus*.

## A surface display system for *S. carnosus*

The first use of recombinant bacteria for surface display of heterologous proteins was first reported more than two decades ago (for reviews, see (Georgiou et al. 1997; Ståhl and Uhlen 1997)) and has since attracted attention for numerous different applications in biotechnology, immunology, and applied microbiology. The first studies were mostly on Gram-negative bacteria, but approaches for surface expression on Gram-positive bacteria soon followed (Samuelson et al. 2002; Ståhl and Uhlen 1997).

**Fig. 3:**
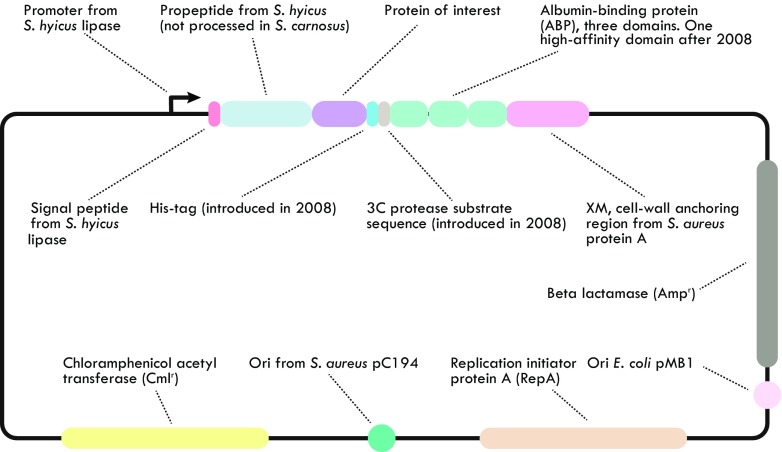
Schematic representation of the expression vector for surface display of recombinant proteins on *S. carnosus*. Please note that the sizes of the different sequence elements are not in scale

In 1995, a novel expression vector for display of recombinant proteins on the surface of *S. carnosus* was described (Samuelson et al. 1995). The vector used the promoter, secretion signal, and propeptide from the *Staphylococcus hyicus* lipase gene in combination with the cell wall anchoring region from staphylococcal protein A (SpA). Between the propeptide and the anchoring part, an albumin-binding protein (ABP), derived from streptococcal protein G, was introduced, enabling efficient monitoring of the surface expression level of individual cells using fluorescently labeled albumin as probe (Fig. 3). In fact, this allowed the quantification of the numbers of heterologously displayed proteins per staphylococcal cell using flow cytometry, and it was assessed that approximately 10^4^ recombinant proteins were displayed per bacterium (Andreoni et al. 1997).

Later, efforts to delete or reduce the size of the propeptide region showed that it in fact was beneficial for display of proteins that were inefficiently secreted (Samuelson et al. 1999). In a successful vector engineering effort, the vector system was further improved in terms of both plasmid size and genetic stability (Wernerus and Ståhl 2002). In addition, the vector system was later modified with a 3C protease substrate sequence (Fig. 3), which enabled specific proteolytic release of displayed proteins to allow detailed characterization (Kronqvist et al. 2008b).

## Miscellaneous early applications for surface-engineered *S. carnosus* cells

### Staphylococcal biocatalysts

Surface display is a straightforward means for production of immobilized enzymes. In a pioneering study, the lipase from *Staphylococcus hyicus* as well as a ß-lactamase from *E. coli* was displayed on *S. carnosus*, and the studies demonstrated that the enzymes retained their catalytic activity (Strauss and Götz 1996). The surface display platform was slightly different compared to the systems described above, with surface-anchoring parts from *S. aureus* fibronectin protein B (FnBPB) instead of the SpA-derived regions. In the study, it was shown that around 10,000 enzymes were displayed on each cell, and the authors also speculated that Gram-positive staphylococci might be particularly appropriate for construction of microbial catalysts due to the rigid cell wall (Strauss and Götz 1996).

### Diagnostic tools

Another interesting application for recombinant bacteria, displaying heterologous proteins on the surface, is so-called whole-cell diagnostic tools. By, for example, displaying antibody fragments or other affinity proteins on the cell, the bacteria could function as “whole-cell monoclonal antibodies” that could be used as diagnostic devises. In a first study on this concept, *S. carnosus* and *S. xylosus* were used for functional surface expression of a murine IgE-specific single-chain variable fragment (scFv) antibody fragment (Gunneriusson et al. 1996). The results from the analysis showed that the recombinant staphylococci could bind to the intended antigen and it was also the first reported display of functional antibody fragments on Gram-positive bacteria. In a follow up study, it was also demonstrated that IgE- and IgA-specific Affibody molecules (see below) could be displayed on *S. carnosus* with retained ability to bind respective antigens (Gunneriusson et al. 1999).

### Directed immobilization of staphylococcal cells

Numerous reports have been published on the concept of surface-specific immobilization of bacteria for different applications, such as for whole-cell biosensors (Scouten 1995), bacterial bioadsorbents (Brower et al. 1997; Kessler 1981), and microbial biocatalysts (Freeman et al. 1996). Specific and directed immobilization of microorganisms to various matrices has the potential to be more straightforward and efficient when compared with conventional strategies, using for example chemical crosslinking, aggregation, or entrapment (Rehm and Omar 2008). In a pioneering study, it was demonstrated that surface expression of a fungal cellulose-binding domain (CBD) from *Trichoderma reesei* cellulase Cel6A on *S. carnosus* resulted in directed immobilization of the bacteria to cellulose fibers (Lehtio et al. 2001).

### Metal-binding staphylococci

Toxic metals in wastewater is a growing issue worldwide and it has been suggested that recombinant bacteria that are displaying metal-binding peptides or proteins might be exploited as bioadsorbents in the bioremediation process (Brower et al. 1997). Another potential application of metal-binding bacteria is in the development of whole-cell microbial biosensors (Tibazarwa et al. 2001). Gram-positive bacteria might have an advantage in terms of bioadsorbents due to the inherent metal-binding capacity of the thick cell wall (Mullen et al. 1989). To investigate the feasibility of the concept, fusion proteins with polyhistidyl peptides for chelation of metal ions were displayed on *S. carnosus* and *S. xylosus* and the results from the study demonstrated that recombinant bacteria could adsorb metal ions as intended (Samuelson et al. 2000).

The promising results on staphylococci as recombinant bioadsorbents inspired additional studies on this application. Instead of using the polyhistidyl peptides as in the previous approach, Wernerus et al. showed that directed evolution by phage display could be used for engineering new Ni^2+^-binding variants of CBD (Wernerus et al. 2001). The isolated CBD variants were subsequently displayed on *S. carnosus*, resulting in recombinant staphylococci with Ni^2+^-binding capacity (Wernerus et al. 2001). The demonstrated ability to generate new specific metal-binding proteins followed by surface expression on staphylococci indicates a potential for straightforward development of inexpensive bioadsorbents for bioremediation of toxic metals in the future.

### *S. carnosus* as a live vaccine delivery system

As mentioned above, *S. carnosus* is a GRAS (generally regarded as safe) organism and has been used extensively in the food industry for decades, which makes it a potentially suitable strain in the vaccine field for oral delivery of recombinant immunogens. In an initial effort, administrations of high doses of *S. carnosus* to mice by mucosal or subcutaneous routes were shown to be safe and well tolerated (Ståhl et al. 1997). Recombinant *S. carnosus* (Samuelson et al. 1995) and *S. xylosus* (Hansson et al. 1992), displaying ABP as a model immunogen, were also used in a comparative immunization study, demonstrating that the *S. carnosus* system was superior compared with *S. xylosus* for oral immunization (Robert et al. 1996; Ståhl et al. 1997). Although the reasons behind these results are not completely clear, the authors speculated that it might be due to the higher surface expression level on *S. carnosus* (Andreoni et al. 1997; Robert et al. 1996).

Although the initial vaccine studies with recombinant staphylococci demonstrated systemic antibody responses, the obtained antibody titers were relatively modest (Liljeqvist and Ståhl 1999). Later investigations were thus focused on strategies for increasing the antibody response to the surface-displayed antigens (Cano et al. 1999; Liljeqvist et al. 1997). The second-generation whole-cell vaccine delivery vehicles were hence modified by fusing the model immunogen to another recombinant protein with adhesive properties, in order to achieve targeting of the bacterial vaccine vehicles to the mucosal epithelium. Three different adhesion proteins were investigated in the study: (i) a fibronectin binding domain from *Streptococcus dysgalactiae* (Liljeqvist et al. 1999), (ii) a cholera toxin B (CTB)-derived peptide, CTBp (Cano et al. 1999), and (iii) a bacterial adhesion factor. The results demonstrated that co-display of any of the evaluated adhesion proteins on the surface of *S. carnosus* yielded elevated serum antibody responses to the displayed immunogen after intranasal administration into mice (Liljeqvist and Ståhl 1999). The encouraging results resulted in a follow-up study, where CTBp was fused to an antigen from the G glycoprotein of human respiratory syncytial virus (RSV) and displayed on *S. carnosus* (Cano et al. 2000). The recombinant staphylococci were thereafter used for intranasal immunization of mice, which elicited a significant anti-RSV serum IgG response. Moreover, lung protection was shown for around 50% of the animals after viral challenge with 100,000 tissue culture infectious doses_50_ (TCID_50_) and was thus the first reported study that could demonstrate protective immunity to a virus using vaccination with recombinant food-grade bacteria (Cano et al. 2000).

## Peptide and protein libraries displayed on *S. carnosus*.

**Fig. 4:**
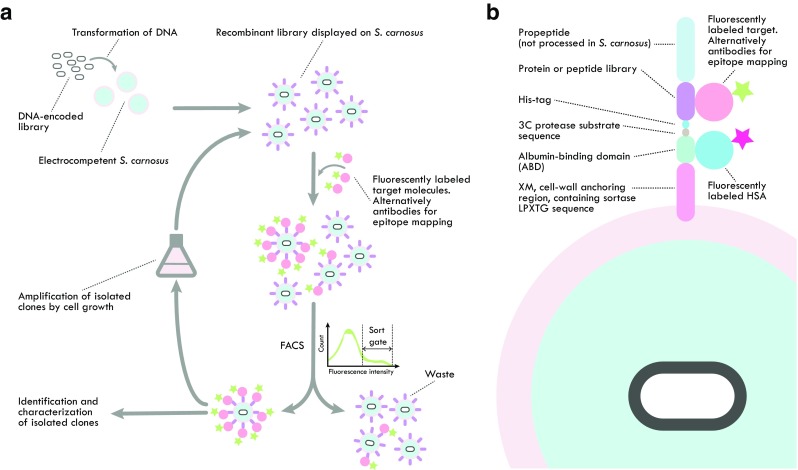
FACS of *S. carnosus* displaying recombinant protein or peptide libraries. **a** Schematic representation of staphylococcal surface display and FACS. Staphylococcal surface expression vectors, encoding protein, or peptide libraries are transformed to *S. carnosus* using electroporation. After expression on the bacterial surface, the combinatorial libraries on staphylococci are incubated with fluorescently labeled target (or antibodies for epitope mapping) and subsequently sorted for isolation of binding variants using FACS. The sorting is typically repeated for several rounds with amplification by growth in between cycles until required enrichment is reached. After sorting, the isolated recombinant proteins or peptides are identified using DNA sequencing. For epitope mapping, the sequence information is used to determine the epitope. For directed evolution of affinity proteins, the affinity as well as the specificity is thereafter determined directly on the cell surface using flow cytometry, followed by subcloning and production of soluble proteins. **b** Schematic representation of the recombinant fusion protein displayed on the surface of *S. carnosus* for library applications and FACS. Cells are incubated with fluorescently labeled target protein (or antibodies for epitope mapping) as well as with fluorescently labeled albumin for monitoring of the surface expression level and normalization during FACS. Please note that approximately 10,000 copies of recombinant protein are displayed per cell, resulting in a quantitative signal in the flow cytometer, corresponding to the affinity for the target

Surface display of recombinant protein and peptide libraries on cells is an attractive complement to the conventional phage display technology. Yeast display of antibody libraries (Cherf and Cochran 2015) is the most established approach, but similar methods have also been developed based on bacteria (Löfblom 2011). The main reason for using cells over phages is the option to use fluorescence-activated cell sorting (FACS) for screening the libraries and for isolation of desired clones (Fig. 4a). The multivalent display of recombinant proteins on the surface (> 10,000 per cell) yields a quantitative fluorescent signal in the flow cytometer that corresponds to the relative affinity, resulting in efficient selection of high affinity variants. Flow-cytometric sorting also provides a direct visualization of the enrichment procedure of binders throughout each selection round in the process.

### Combinatorial protein engineering of affinity proteins using staphylococcal display and FACS

The first study showing the potential of *S. carnosus* for combinatorial protein engineering was published in 2003 and demonstrated that recombinant Affibody molecules (Löfblom et al. 2010; Nord et al. 1997; Ståhl et al. 2017) displayed on the bacterial surface could be enriched by FACS from a large background (1:100,000) of non-binders (Wernerus et al. 2003).

One of the characteristics of cell display of combinatorial libraries combined with FACS is the ability to discriminate between variants with relatively small differences in affinity, facilitating isolation of the strongest binders during selection. In a study from 2005, a mock sorting was conducted to explore the discrimination capacity of the staphylococcal method (Löfblom et al. 2005). The albumin-binding fusion protein was employed as a surface expression monitoring tag and cells were labeled with a saturating concentration of fluorescently labeled albumin (Fig. 4b). By normalizing the target-binding signal with the surface-expressing level, the distribution in signal was reduced dramatically. It was furthermore demonstrated that flow-cytometric sorting from a mock library on *S. carnosus* that contained binders with twofold higher affinity mixed 1:1000 in a background of a weaker variant resulted in efficient isolation from a single round with an enrichment factor of around 140-fold.

Another feature of cell display is that isolated individual variants after sorting can be characterized directly on the cell surface using flow cytometry, obviating initial subcloning for soluble protein production of candidates. Using the staphylococcal display platform, it was demonstrated that both the equilibrium dissociation constant and the dissociation rate constant could be accurately determined in the flow cytometer (Löfblom et al. 2007b). Moreover, in a following publication, it was shown that the recombinant protein could be released from the surface by specific proteolytic cleavage and that the obtained soluble binders were functional in different assays (Kronqvist et al. 2008b).

Although the studies described so far had shown the potential of *S. carnosus* for library applications, the most critical challenge still remained—transformation of the DNA-encoded library to the staphylococcal host. While the relatively thick peptidoglycan cell wall is a favorable feature in FACS as the viability of isolated clones is nearly unaffected by the harsh sorting conditions, it also limits the transformation frequency. Library complexity and the probability of finding high-affinity variants are directly correlated, which means that transforming millions of clones is basically a requirement for success in combinatorial protein engineering. In an effort to increase the relatively modest transformation frequency of *S. carnosus,* a number of different parameters for electroporation were optimized, including the addition of a heat-treatment step to temporarily knock out the host restriction enzymes, enabling transformation of high concentrations of plasmid DNA prepared in *E. coli* (Löfblom et al. 2007a). Overall, the optimization resulted in 10,000-fold higher transformation frequency, corresponding to around 10^6^ transformed staphylococci per electroporation event.

The improved transformation frequency opened up the possibility to construct large libraries on *S. carnosus*. In a pioneering study, a pre-selected Affibody library from phage display was transferred to staphylococci and subnanomolar binders for tumor necrosis factor (TNF) alpha; TNF alpha were efficiently isolated using FACS (Kronqvist et al. 2008a). Following the first reported staphylococcal library, the method has since been used extensively for affinity maturation of Affibody molecules. Examples include affinity maturation of a human epidermal growth factor receptor 3 (HER3)-specific Affibody down to around 20 pM affinity (Kronqvist et al. 2011; Malm et al. 2013), affinity maturation of a head-to-tail dimeric Affibody for the amyloid beta peptide to 300 pM affinity (Lindberg et al. 2013, 2015), and more recently, affinity maturation of two distinct Affibody molecules for vascular endothelial growth factor receptor 2 (VEGFR2) (Fleetwood et al. 2014), which were later formatted as a so-called biparatopic binder with extremely slow dissociation from the receptor (Fleetwood et al. 2016).

In addition to combinatorial engineering of Affibody molecules, the method has also been used for isolation of other types of affinity proteins. One example is engineering of so-called ADAPT molecules (ABD-derived affinity protein), which are based on an albumin-binding domain from streptococcal protein G (Alm et al. 2010). For the ADAPTs, the libraries were designed with the intention of preserving the affinity for albumin, while engineering an additional specific binding on the opposite surface of the affinity protein. In these efforts, the possibility to use multiparameter FACS was exploited and albumin and target were labeled with different fluorophores, enabling efficient engineering of bispecific binders by monitoring both signals simultaneously in the flow cytometer. Bispecific ADAPTs for TNF (Nilvebrant et al. 2011), human epidermal growth factor receptor 2 (HER2) (Nilvebrant et al. 2014), and HER3 (Åstrand 2016), respectively, have been successfully isolated using that approach from libraries displayed on staphylococci.

Staphylococcal display has also been used for selection of specific antibody fragments. Fleetwood and coworkers subcloned an immune so-called nanobody (i.e., single-domain VHH from camelid heavy-chain-only antibodies) library to the staphylococcal display vector and used FACS for isolation of green fluorescent protein (GFP)-specific camelid antibodies (Fleetwood et al. 2013). The same library had previously been used for selecting nanobodies to GFP using phage display and when comparing the output from the two methods, it was demonstrated that the staphylococcal method yielded binders with a higher affinity on average and that the clones were relatively different between the two methods. Another more recent example is a study where *S. carnosus* was used for engineering of HER2-specific single-chain variable fragment (scFv) antibodies (personal communication Johan Rockberg, KTH). Staphylococcal display and FACS was used both for selection of first-generation binders as well as for affinity maturation with an error-prone PCR-generated library to yield human scFvs in the low nanomolar range.

### Display of peptide libraries on *S. carnosus* for profiling of protease substrates

In addition to the generation of specific affinity proteins, the staphylococcal platform has been evaluated for display of peptide libraries for various purposes. One recent publication describes how the method can be utilized for substrate profiling of proteases as well as for discovery of new improved substrates that are processed with higher catalytic activity. The method is based on display of random peptide substrate libraries on the surface of staphylococci followed by addition of protease and subsequent FACS for isolation and identification of cleaved substrates. In the method, an Affibody is expressed on the surface as a reporter tag. In-fusion with the reporter tag is another domain that specifically blocks the reporter tag from binding a soluble fluorescently labeled reporter (Sandersjoo et al. 2015). The linker between the two domains contains a substrate peptide library. Upon addition of protease, variants with a functional substrate will be cleaved within the linker, resulting in release of the blocking domain and binding of the fluorescent reporter molecule. Using substrate libraries for tobacco etch virus (TEV) protease and matrix metalloprotease (MMP)-1, the substrate profiles for the respective protease were identified and several new peptides were isolated for MMP-1 that were processed with up to eightfold higher catalytic activity compared with previously reported substrates (Sandersjoo et al. 2017).

### Display of peptide and protein libraries on *S. carnosus* for epitope mapping

Several studies have also reported on the use of the staphylococcal display method for epitope mapping of antibodies. Two approaches have so far been explored for this purpose. The first is based on surface display of antigen-derived peptide libraries, differing in length and covering the entire sequence of the antigen. By incubating the peptide libraries with fluorescently labeled antibodies and subsequent FACS (Fig. 4), the corresponding epitopes for both monoclonal and polyclonal antibodies binding to a panel of different antigens have been identified (Hjelm et al. 2010, 2012; Kronqvist et al. 2010; Rockberg et al. 2008, 2010). In a more large-scale approach, Hudson and colleagues created a peptide library covering the sequences of 60 clinically relevant protein targets (Hudson et al. 2012). The library was used to map the epitopes of several different antibodies and sequencing the output revealed off-target binding in some cases, demonstrating that the strategy is also powerful for investigating potential cross-reactivity. Although the lengths of the peptides in the library can be adjusted and it has been shown that conformational epitopes might be identified for certain antigen/antibody pairs, in general, the antigen-derived peptide libraries are more suitable for discovery of linear epitopes. Another complementary approach is to express full-length proteins or independently folded domains and construct error-prone PCR libraries on staphylococci followed by sorting for loss of binding. This strategy was used for mapping the conformational epitope of the monoclonal antibody eculizumab, which is used in the clinics for treating patients with paroxysmal nocturnal hemoglobinuria (PNH) and atypical hemolytic uremic syndrome (aHUS) (Volk et al. 2016). Interestingly, the identified epitope explained the previously observed non-responsiveness to treatment in a subpopulation of patients of Japanese origin, carrying a mutation in the epitope.

## Conclusions and future perspectives

*S. carnosus* is a non-pathogenic Gram-positive staphylococcal species. It has for a long time (and is still today) been used as part of starter cultures for meat fermentation and in other food processes. An essential function of *S. carnosus* in starter cultures is to prevent the growth of undesirable bacteria, thus reducing the risk of food poisoning and acting as a food preservative. Importantly, *S. carnosus* also contributes favorably to development of flavor and red color as well as to decreasing pH and hydrogen peroxide. Due to the many valuable and often unique properties of *S. carnosus*, it will most likely continue to play an important role in food processing in the future. In 2009, the genome sequence of *S. carnosus* was published, which verified and explained its previously reported non-pathogenic behavior. *S. carnosus* lacks important virulence factors found in many pathogenic bacteria. This has made *S. carnosus* a very valuable scientific model organism for studying and elucidating the mechanism of isolated genes from, for example, *S. aureus* for pathogenicity. As staphylococcal infections and the general issue of increasing antibiotic resistance is continuing to grow globally, we expect that *S. carnosus* will be an even more important tool for such studies in the future, as part of the large efforts to combat these, sometimes deadly, infectious diseases. Due to the long historic use in the food industry and the now verified non-pathogenic properties, *S. carnosus* is classified as a GRAS organism. Moreover, the straightforward translocation of recombinant proteins over the single-cell membrane in Gram-positive bacteria combined with the very low proteolytic extracellular activity makes *S. carnosus* an attractive host for production of secreted recombinant proteins. Methods for transformation, subcloning, and protein production in *S. carnosus* are established today and yields of grams per liter culture for recombinant human proteins have been reported. Although the post-translational modifications of human proteins are different compared to eukaryotic hosts, *S. carnosus* has the potential to become an attractive complementary prokaryotic production host in cases when such modifications are not critical for the intended application.

In addition to secreted production of soluble recombinant proteins, a vector system has also been developed for surface display of recombinant proteins and peptides on the surface of *S. carnosus*. It has been used for a number of different applications, such as display of metal-binding peptides with the long-term goal of using them as whole-cell bioadsorbents for purification of metal pollutants from wastewater. Another example is a whole-cell biocatalyst where enzymes are displayed on the bacteria, obviating the need for production and purification of soluble enzymes. Since *S. carnosus* is a GRAS organism and not pathogenic, it has also been investigated relatively extensively as a vaccine delivery vehicle, carrying antigenic determinants displayed on the surface, in several preclinical vaccination studies with encouraging results. More recently, optimization of the DNA transformation efficiency has enabled expression of large libraries of recombinant proteins or peptides on *S. carnosus*. Screening such libraries with FACS has been used for directed evolution of a range of different affinity proteins, substrate profiling of proteases, as well as for epitope mapping of antibodies. With the successful results from these different library applications, we expect that *S. carnosus* will be a valuable complement to phage and yeast display in the years to come.

In summary, *S. carnosus* will definitely continue to be an important microorganism in a very broad range of applications in the future, all the way from being part of starter cultures in sausage fermentation to host in powerful methods for directed evolution of new biopharmaceuticals.
